# Numerical Modeling of Load-Driven Changes in Squat Technique Using a Moment-Limited Joint Framework

**DOI:** 10.3390/bioengineering13050485

**Published:** 2026-04-22

**Authors:** Karol Nowak, Anna Szymczak-Graczyk, Aram Cornaggia, Tomasz Garbowski

**Affiliations:** 1 Faculty of Education Studies, Kazimiera Milanowska College of Education and Therapy, 61-473 Poznan, Poland; k.nowak@wseit.edu.pl; 2 Faculty of Environmental and Mechanical Engineering, Poznan University of Life Sciences, 60-637 Poznan, Poland; anna.szymczak-graczyk@up.poznan.pl; 3 Department of Engineering and Applied Sciences, Università degli Studi di Bergamo, 24044 Dalmine, BG, Italy; aram.cornaggia@unibg.it; 4 University Center for Ecomaterials, Poznan University of Life Sciences, 60-637 Poznan, Poland

**Keywords:** squat biomechanics, barbell load, joint moment capacity, nonlinear joint mechanics, nonlinear optimization, predictive simulation, load-dependent posture adaptation, computational biomechanics

## Abstract

The squat is a fundamental multi-joint movement widely studied in strength training and biomechanics. While numerous experimental and computational studies have examined squat kinematics and joint loading, the mechanisms governing how squat technique adapts to increasing external load remain insufficiently understood. In particular, inverse-dynamics-based approaches often overlook explicit constraints imposed by limited joint moment capacity. This study presents a computational framework for predicting load-dependent adaptations of squat posture. The human body was represented as a multi-segment rigid-body system, with joints modeled as nonlinear rotational elements with bounded moment capacity. A reference squat trajectory was first generated kinematically, and a constrained optimization procedure was then applied at each motion frame to determine a mechanically admissible posture under increasing barbell load. The results show that higher loads lead to systematic posture adaptations, including increased torso inclination and redistribution of rotational demand from the knee toward the hip joint. For the highest load, peak torso pitch increased from 30° to over 40°, while joint utilization exceeded unity, indicating the onset of yielding. These findings identify joint moment capacity as a key constraint governing squat technique and demonstrate the potential of the proposed framework for predictive biomechanical analysis.

## 1. Introduction

The squat is one of the most fundamental multi-joint movements in human locomotion and strength training, widely used in both athletic performance and rehabilitation contexts [1,2]. Its biomechanical complexity arises from the coordinated interaction of multiple body segments, including the lower limbs, pelvis, and torso, which must adapt continuously to maintain balance and mechanical efficiency under varying loading conditions [3,4,5].

In addition to its relevance for performance training, the squat has also been widely used as a model task in human movement research to investigate lumbopelvic coordination, lower-limb mechanics, and trunk control under load. Previous experimental studies have shown that squat execution depends strongly on segment coordination and on the ability to maintain balance while redistributing joint demand across the ankle, knee, hip, and trunk. These observations indicate that squat mechanics should be interpreted not only kinematically, but also in terms of the underlying mechanical constraints that shape feasible movement strategies.

A substantial body of research has investigated how variations in squat technique influence joint kinematics, kinetics, and muscle activation patterns. Key factors such as stance width, bar position, and movement strategy have been shown to significantly affect load distribution across the hip, knee, and ankle joints [6,7,8]. In particular, different squat styles, including traditional, powerlifting, and box squat techniques, exhibit distinct movement patterns characterized by varying degrees of knee displacement, hip backshift (defined here as the posterior displacement of the pelvis center relative to its initial position), and torso inclination [9,10,11].

One of the most frequently discussed aspects of squat mechanics is the role of knee translation. Limiting anterior knee displacement has been associated with reduced knee joint loading but increased demands on the hip extensors, resulting in modified joint torque distributions and changes in trunk posture [12,13]. These findings highlight the inherent trade-offs between joint loading mechanisms and movement strategies.

Beyond kinematics, numerous studies have explored the relationship between squat execution and performance-related variables such as force production, velocity, and power output [14,15,16]. In particular, the rate of force development and peak power have been identified as key indicators of athletic performance, although their dependence on technique and loading conditions remains complex [17]. Additionally, musculotendinous stiffness and the interaction between eccentric and concentric phases have been shown to influence movement efficiency and force transmission [18].

Experimental investigations have further demonstrated that squat technique affects not only performance metrics but also potential injury mechanisms. Variations in joint alignment, particularly involving hip internal/external (axial) rotation and knee valgus, may increase stress on the knee joint and contribute to injury risk [19,20]. Accurate measurement and modeling of body kinematics and center-of-mass motion are therefore essential for reliable biomechanical analysis [21,22].

Despite these extensive experimental efforts, most existing studies rely on inverse dynamics approaches, in which joint moments are calculated from measured motion and external forces. While this methodology provides valuable insight into the mechanical consequences of observed movement, it does not explicitly address whether a given motion is mechanically admissible under realistic joint strength limitations [23,24,25].

From a mechanics perspective, human motion can be interpreted as the result of competing constraints imposed by geometry, external loading, and internal strength capacity. In this context, joint moment limits may play a governing role in shaping movement strategies, particularly under large external loads [26,27,28]. However, this aspect remains relatively underexplored in current biomechanical modeling frameworks.

This gap is particularly relevant in the context of predictive simulation and mechanically constrained movement modeling. While musculoskeletal and inverse dynamics frameworks have greatly improved the analysis of observed motion, they are often used to evaluate motion that has already occurred rather than to predict how posture may change when mechanical limits become active. For loaded multi-joint tasks such as the squat, this distinction is important because the performed technique may emerge from a trade-off between movement intent, balance requirements, and the finite strength of the involved joints.

Recent developments in computational biomechanics suggest that integrating kinematic modeling with mechanical constraints may provide a more complete description of human motion [29,30]. Such approaches enable the investigation of how movement patterns emerge as a result of mechanical feasibility rather than being prescribed a priori.

In this study, a computational framework was developed for reconstructing squat motion under external loading while explicitly accounting for joint moment capacity. The method combined kinematic trajectory generation with a nonlinear joint model and a constrained optimization procedure, allowing the motion to adapt dynamically to mechanical limitations.

The novelty of the proposed approach lies in the explicit integration of bounded joint moment capacity into a predictive frame-by-frame posture correction scheme, so that technique adaptation is not prescribed a priori but emerges from the interaction between tracking objectives, external loading, and mechanical feasibility. In this way, the framework complements classical inverse dynamics by addressing not only the consequences of an observed movement, but also the mechanical admissibility of alternative movement strategies under load.

The objective of the present study was therefore to investigate how increasing barbell load modifies squat posture and redistributes joint demand when joint strength limits are treated as active biomechanical constraints.

## 2. Materials and Methods

The squat motion was modeled using a three-dimensional, segment-based representation of the human body. The model consisted of rigid segments corresponding to the feet, shanks, thighs, pelvis, and torso, connected by rotational joints. The formulation involved a reduced set of generalized coordinates describing the global posture of the system, while intermediate joint positions were obtained through geometric constraints.

The model was implemented in a custom computational environment (MATLAB (R2024a)-based), and all simulations were performed using a frame-by-frame quasi-static approach. The total number of active degrees of freedom in the mechanical correction stage was five, corresponding to pelvis translation, pelvis orientation, and torso inclination.

The motion was parameterized through a reduced set of generalized coordinates describing the position and orientation of the pelvis, as well as the inclination of the torso. These variables defined the global posture of the system, while the positions of intermediate joints, such as knees and hips, were determined through geometric constraints ensuring compatibility with prescribed segment lengths. This formulation enabled the generation of smooth squat trajectories while preserving kinematic consistency of the lower-limb mechanism.

**Figure 1:**
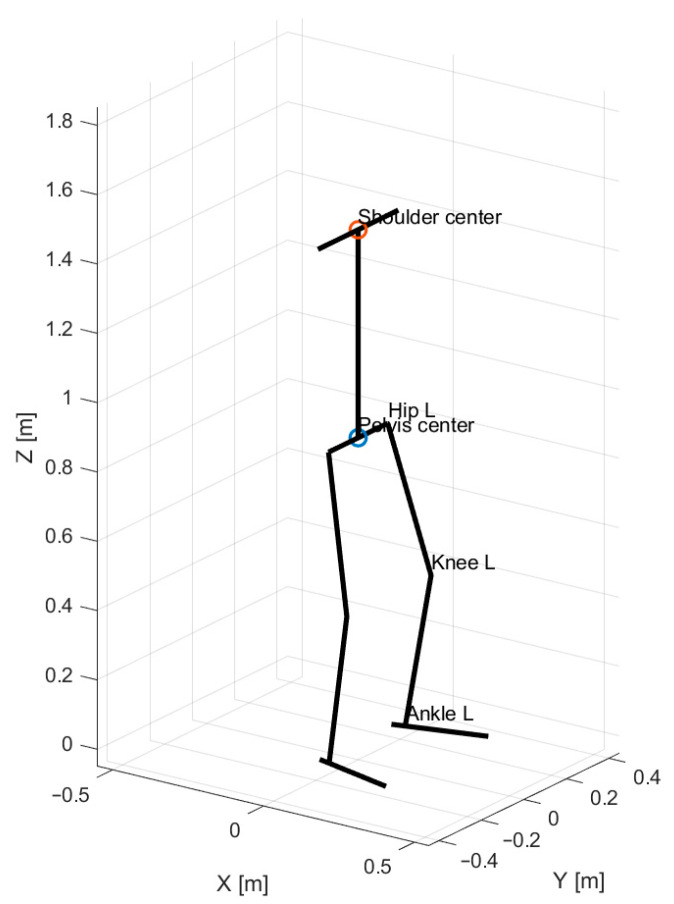
Three-dimensional biomechanical model used in the study, including segment definitions, joint locations, and kinematic variables governing squat motion.

The adopted three-dimensional kinematic representation, including the definition of body segments, joint locations, and principal geometric variables, is illustrated in Figure 1. In this representation, the pelvis center is defined as the midpoint between the left and right hip joint centers.

The reference motion was generated using a smooth parametric profile, which controlled the vertical displacement and horizontal shift of the pelvis, as well as the progressive inclination of the torso. This reference trajectory served as a baseline kinematic solution that did not account for mechanical limitations of the joints. In subsequent steps, the motion was corrected through an optimization procedure that enforced joint moment constraints and allowed for load-dependent modifications of posture.

To this end, each joint was modeled using a nonlinear rotational element with bounded moment capacity, enabling the representation of elastic behavior followed by gradual stiffness reduction once a critical moment level was approached. This formulation provided a continuous transition between elastic- and plastic-like response, allowing the model to capture the onset of mechanical limitations without introducing discontinuities in the solution.

The anthropometric parameters used in the model were selected to represent a typical adult male subject and were based on commonly reported proportions in the biomechanics literature. Segment lengths and body proportions were chosen to ensure realistic kinematic behavior, while maintaining a simplified and generic representation suitable for parametric analysis.

### 2.1. Kinematic Representation of the Body

The model was defined in a global Cartesian coordinate system $\left(X,Y,Z\right)$, where the X-axis denotes the anterior–posterior direction, the Y-axis corresponds to the left–right direction, and the Z-axis is vertical. The body was represented by rigid segments corresponding to the feet, shanks, thighs, pelvis, and torso. The left and right legs were treated separately, while the pelvis and shoulder girdle were modeled as horizontal connecting segments.

The generalized coordinates used for the mechanical correction step were collected in the vector

(1) $$q={\left[\begin{array}{ccccc} x_{p} & z_{p} & \psi_{p} & \phi_{p} & \theta_{t} \end{array}\right]}^{T},$$

where $x_{p}$ and $z_{p}$ are the horizontal and vertical coordinates of the pelvis center, $\psi_{p}$ is the pelvis rotation about the global Z-axis, $\phi_{p}$ is the pelvic obliquity (rotation about the global *X*-axis), and $\theta_{t}$ is the torso pitch angle (rotation about the global *Y*-axis, i.e., in the sagittal plane). In this formulation, the *X*-axis corresponds to the frontal axis, the *Y*-axis to the sagittal axis, and the *Z*-axis to the vertical direction. The adopted coordinate vector provides a compact description of the global posture of the body. In particular, $x_{p}$ controls the horizontal position of the pelvis relative to the fixed foot positions, $z_{p}$ defines squat depth, $\psi_{p}$ represents axial rotation of the pelvis, $\phi_{p}$ describes pelvic obliquity (rotation in the frontal plane), and $\theta_{t}$ governs the forward inclination of the torso (sagittal-plane rotation). Together, these variables capture the primary degrees of freedom involved in load-dependent posture adaptation.

The lateral translation of the pelvis center was neglected in the present study, which is consistent with the assumed dominant symmetry of the movement in the frontal direction and with the adopted motion drivers.

For a given q, the center of the pelvis was defined as

(2) $$p_{p}=\left[\begin{array}{c} x_{p}\\ 0\\ z_{p} \end{array}\right].$$

The pelvis orientation was described by the rotation matrix

(3) $$R_{p}=R_{z}\left(\psi_{p}\right)R_{x}\left(\phi_{p}\right),$$

where $R_{z}(\psi_{p})$ and $R_{x}(\phi_{p})$ are the standard rotation matrices about the Z- and X-axes, respectively.

(4) $$R_{z}\left(\psi_{p}\right)=\left[\begin{array}{ccc} \cos\psi_{p} & -\sin\psi_{p} & 0\\ \sin\psi_{p} & \cos\psi_{p} & 0\\ 0 & 0 & 1 \end{array}\right],\;\;\;\;\;R_{x}\left(\phi_{p}\right)=\left[\begin{array}{ccc} 1 & 0 & 0\\ 0 & \cos\phi_{p} & -sin\phi_{p}\\ 0 & \sin\phi_{p} & \cos\phi_{p} \end{array}\right].$$

The variable $b_{p}$ denotes the pelvis width. The local transverse unit vector of the pelvis was obtained from

(5) $$e_{y,p}=R_{p}\left[\begin{array}{c} 0\\ 1\\ 0 \end{array}\right]$$

and the hip joint centers were then written as

(6) $$p_{H_{L}}=p_{p}+\frac{b_{p}}{2}e_{y,p},\;\;\;\;\;p_{H_{R}}=p_{p}-\frac{b_{p}}{2}e_{y,p}.$$

The ankles were assumed to be fixed in space throughout the motion, which corresponded to a stance with prescribed foot positions. Thus,

(7) $$p_{A_{L}}=p_{A_{L}}^{0},\;\;\;\;\;p_{A_{R}}=p_{A_{R}}^{0},$$

where $p_{A_{L}}^{0}$ and $p_{A_{R}}^{0}$ denote the initial coordinates of the left and right ankle joints. The toes and heels were reconstructed from the ankle locations and the prescribed foot orientation angles, but since the presented mechanical model was driven primarily by pelvis and torso variables, these points were used mainly for visualization and interpretation rather than as additional mechanical degrees of freedom.

### 2.2. Local Leg Planes and Inverse Kinematics

Each leg was solved in its own vertical plane. This construction was important because it allowed the left and right limbs to differ in orientation while avoiding the complexity of a full three-dimensional inverse kinematics formulation. $\alpha_{L}$ and $\alpha_{R}$ denote the yaw angles of the left and right feet in the horizontal plane. The corresponding unit vectors defining the local anterior direction of each leg were given by

(8) $$e_{x,L}=\left[\begin{array}{c} \cos\alpha_{L}\\ \sin\alpha_{L}\\ 0 \end{array}\right],\;\;\;\;\;e_{x,R}=\left[\begin{array}{c} \cos\alpha_{R}\\ \sin\alpha_{R}\\ 0 \end{array}\right],$$

whereas the local vertical direction was common for both legs,

(9) $$e_{z}=\left[\begin{array}{c} 0\\ 0\\ 1 \end{array}\right].$$

For each side, the hip position was projected onto the corresponding local sagittal plane attached to the ankle. For the left leg, the local coordinates of the hip were

(10) $$x_{H_{L}}=\left(p_{H_{L}}-p_{A_{L}}\right)\cdot e_{x,L},\;\;\;\;\;z_{H_{L}}=\left(p_{H_{L}}-p_{A_{L}}\right)\cdot e_{z},$$

and analogously for the right leg,

(11) $$x_{H_{R}}=\left(p_{H_{R}}-p_{A_{R}}\right)\cdot e_{x,R},\;\;\;\;\;z_{H_{R}}=\left(p_{H_{R}}-p_{A_{R}}\right)\cdot e_{z}.$$

$L_{s}$ and $L_{f}$ denote the lengths of the shank and thigh, respectively. For each leg, the knee position was obtained as the intersection of two circles in the local plane: one centered at the ankle with radius $L_{s}$, and the other centered at the hip with radius $L_{f}$. Thus, for the left leg, the local knee coordinates $\left(x_{K_{L}},z_{K_{L}}\right)$ satisfied

(12) $$x_{K_{L}}^{2}+z_{K_{L}}^{2}=L_{s}^{2},$$

(13) $$(x_{H_{L}}-x_{K_{L}}{)}^{2}+(z_{H_{L}}-z_{K_{L}}{)}^{2}=L_{f}^{2},$$

and the same form was used for the right leg. The admissibility of the inverse kinematics solution required

(14) $$\left|L_{s}-L_{f}\right|\leq \sqrt{x_{H_{i}}^{2}+z_{H_{i}}^{2}}\leq L_{s}+L_{f},\;\;\;\;\;\;\;\;\;\;i\in \left\{L,R\right\},$$

which defined the reachable domain of the hip relative to the ankle. Once the local knee coordinates were found, the three-dimensional position of the knee was reconstructed as

(15) $$p_{K_{i}}=p_{A_{i}}+x_{K_{i}}e_{x,i}+z_{K_{i}}e_{z},\;\;\;\;\;\;\;\;\;\;\;i\in \left\{L,R\right\}.$$

This formulation preserved the exact lengths of the thigh and shank and ensured that the kinematic correction of the upper body remained compatible with the lower-limb geometry.

### 2.3. Torso and Shoulder Girdle

The torso was modeled as a rigid segment of length $L_{t}$ connecting the pelvis center and the shoulder center. If the torso was assumed to follow the pelvis yaw, then its horizontal projection was aligned with the rotated sagittal direction of the pelvis. The torso direction vector was therefore written as

(16) $$d_{t}=\left[\begin{array}{c} \sin\theta_{t}\cos\psi_{p}\\ \sin\theta_{t}\sin\psi_{p}\\ \cos\theta_{t} \end{array}\right]$$

and the shoulder center became

(17) $$p_{S_{c}}=p_{p}+L_{t}\;d_{t}.$$

$b_{s}$ denotes the shoulder width. The left and right shoulder points were then obtained from

(18) $$p_{S_{L}}=p_{S_{c}}+\frac{b_{s}}{2}e_{y,s},\;\;\;\;\;p_{S_{R}}=p_{S_{c}}-\frac{b_{s}}{2}e_{y,s},$$

where $e_{y,s}$ is the shoulder transverse direction. In the present implementation, this direction followed the pelvis yaw, which provided a geometrically consistent upper-body representation without introducing additional rotational degrees of freedom.

The torso was modeled as a single rigid segment, neglecting internal spinal articulation. This simplification was adopted to reduce model complexity and to focus the analysis on global posture adaptation rather than local spinal mechanics. As a result, the model did not capture detailed load distribution within the lumbar region, which represents a limitation of the present formulation.

### 2.4. Reference Trajectory

The reference squat motion was generated by prescribing smooth trajectories for the pelvis center and torso orientation. Specifically, the pelvis descended vertically and shifted posteriorly, while the forward torso pitch (i.e., flexion in the sagittal plane) increased with squat depth. s∈[0,1] denotes the normalized motion parameter, where s = 0 corresponds to the initial upright position and s=1 to the deepest squat position. A smooth profile function $f\left(s\right)$ was introduced as

(19) $$f(s)=3s^{2}-2s^{3},$$

which guaranteed zero slope at both endpoints and therefore avoided abrupt transitions between frames. The smooth step function was selected due to its $C_{1}$ continuity, ensuring zero velocity at the beginning and end of the motion. This avoided artificial acceleration discontinuities and provided a physically consistent transition between successive frames.

Using the maximum amplitudes of the prescribed motion, the reference generalized coordinates were defined as

(20) $$x_{p}^{ref}(s)=x_{p,0}-\Delta x_{p}\;f(s),$$

(21) $$z_{p}^{ref}(s)=z_{p,0}-\Delta z_{p}\;f(s)$$

(22) $$\psi_{p}^{ref}\left(s\right)=\psi_{p,max}f\left(s\right),\;\;\;\;\;\phi_{p}^{ref}\left(s\right)=\phi_{p,max}f\left(s\right),\;\;\;\;\;\theta_{t}^{ref}(s)=\theta_{t,max}f(s),$$

where $x_{p,0}$ and $z_{p,0}$ denote the initial pelvis coordinates, and $\Delta x_{p}$, $\Delta z_{p}$, $\psi_{p,max}$, $\phi_{p,max}$, and $\theta_{t,max}$ are user-defined amplitudes. The reference motion did not yet account for the effect of external loading or joint strength limits; instead, it represented the intended squat pattern to be corrected in the next step.

### 2.5. Joint Variables and Local Angular Measures

The purpose of the mechanical correction was not to model beam bending in the body segments, but to capture the rotational demand in the principal joints. For this reason, the body segments were treated as rigid, while the joints were represented by nonlinear rotational elements. The relevant angular quantities were extracted from the reconstructed geometry.

For each leg, the shank and thigh orientations in the local plane were determined from the corresponding segment vectors. The local shank angle was obtained from the ankle-to-knee direction, and the local thigh angle from the knee-to-hip direction. Based on these, the ankle, knee, and hip joint measures were introduced in a form consistent with the adopted computational implementation. For the left side,

(23) $$\theta_{a,L}=\theta_{s,L},$$

(24) $$\theta_{k,L}=\theta_{s,L}-\theta_{f,L},$$

(25) $$\theta_{h,L}=\theta_{f,L}-\phi_{p},$$

where $\theta_{s,L}$ is the shank angle and $\theta_{f,L}$ is the thigh angle in the local left-leg plane. Analogous definitions were used for the right side. The torso rotational measure was defined as the relative angle between the torso and the pelvis tilt:

(26) $$\theta_{\tau }=\theta_{t}-\phi_{p}.$$

These angular measures were evaluated both for the reference motion and for the mechanically corrected posture. Their differences drove the joint moments and energy contributions. In the present formulation, joint moments are evaluated about the medio-lateral axis and therefore correspond to flexion–extension behavior in the sagittal plane.

### 2.6. Nonlinear Joint Model with Moment Capacity

The mechanical behavior of each joint was described using a bounded nonlinear moment–rotation law. $\Delta \theta_{j}$ denotes the deviation of the current joint angle from its reference value:

(27) $$\Delta \theta_{j}=\theta_{j}-\theta_{j}^{ref},$$

where j denotes one of the active joints: left ankle, left knee, left hip, right ankle, right knee, right hip, or torso. The restoring moment in joint j was then defined as

(28) $$M_{j}\left(\Delta \theta_{j}\right)=M_{y,j}\tanh\left(\frac{k_{j}\;\Delta \theta_{j}}{M_{y,j}}\right),$$

where $k_{j}$ is the active rotational stiffness and $M_{y,j}$ is the critical moment capacity. This expression had two desirable properties. First, for small angular deviations, it recovered an approximately linear elastic response:

(29) $$M_{j}\left(\Delta \theta_{j}\right)\approx k_{j}\Delta \theta_{j}\;\;\;\mathrm{for}\;\;\;|\Delta \theta_{j}|\ll 1.$$

Second, for large deviations, it asymptotically approaches the joint capacity:

(30) $$\left|M_{j}\left(\Delta \theta_{j}\right)\right|\to M_{y,j}\;\;\;\;\;\;\;\;\;\;\;as\;\;\;\;\;\;\;\;\;\;\;\left|\Delta \theta_{j}\right|\to \infty ,$$

which regularizes the response and avoids nonphysical unlimited moment growth. This choice was made deliberately to obtain a smooth constitutive model suitable for frame-by-frame optimization.

The energy associated with joint j was obtained by integrating Equation (28), which yielded

(31) $$\Pi_{j}\left(\Delta \theta_{j}\right)=\frac{M_{y,j}^{2}}{k_{j}}\ln\left(\cosh\left(\frac{k_{j}\;\Delta \theta_{j}}{M_{y,j}}\right)\right).$$

The total joint contribution to the objective function was then computed as the sum over all active joints:

(32) $$\Pi_{joint}=\sum_{j\in J}\Pi_{j}\left(\Delta \theta_{j}\right),$$

where J was the set of seven modeled rotational components.

The joint moment capacities used in the model were selected based on representative values reported in the biomechanics literature and scaled to reflect relative strength differences between joints. The adopted values should be interpreted as indicative rather than subject-specific, as the focus of the study is on the qualitative behavior of the system.

### 2.7. Yield Criterion and Stiffness Degradation

A key assumption of the present study was that the posture may change when the rotational demand in a joint approaches its strength limit. Instead of introducing a full incremental plasticity model with explicit plastic rotations, a simpler but robust mechanism was adopted. Once the moment in a given joint reached a prescribed fraction of its capacity, the joint was considered yielded and its stiffness was permanently reduced in subsequent frames.

The yield condition was written as

(33) $$\left|M_{j}\right|\geq \eta_{y}\;M_{y,j},$$

where $\eta_{y}\in (0,1)$ is the yield ratio used for numerical stability. After yielding, the active stiffness was updated according to

(34) $$k_{j}\leftarrow k_{p,j},$$

where $k_{p,j}\ll k_{e,j}$, and $k_{e,j}$ denotes the initial elastic stiffness. In the present implementation, the reduced stiffness $k_{p,j}$ was set to approximately 5–10% of the initial elastic stiffness $k_{e,j}$, ensuring a significant but numerically stable reduction in joint resistance after yielding.

This mechanism represented a phenomenological softening of the joint once its mechanical limit was reached. Although simplified, it was sufficient to investigate how strength limitations may altered the squat posture under increasing external load.

### 2.8. External Loading from the Barbell

The external load was represented by a barbell of mass $m_{b}$, assumed to act vertically downward through the shoulders. The total barbell weight was

(35) $$W_{b}=m_{b}g,$$

where g is gravitational acceleration. In the present formulation, the barbell load was distributed equally between the left and right shoulder points. Since the model was formulated through an energy-based objective, the gravitational contribution was introduced as the potential of the external forces:

(36) $$\Pi_{ext}=\frac{1}{2}W_{b}\;z_{S_{L}}+\frac{1}{2}W_{b}\;z_{S_{R}},$$

where $z_{S_{L}}$ and $z_{S_{R}}$ are the vertical coordinates of the left and right shoulders. This term drove the mechanically corrected posture toward lower potential energy while competing with the reference-motion tracking and the resistance of the joints.

### 2.9. Tracking Term and Total Objective Function

The mechanically corrected squat was assumed to remain close to the intended kinematic motion. This was enforced through a quadratic tracking term:

(37) $$\Pi_{track}=\frac{1}{2}{\left(q-q^{ref}\right)}^{T}K_{track}\left(q-q^{ref}\right),$$

where $q^{ref}$ is the generalized-coordinate vector obtained from the reference motion and $K_{track}$ is a diagonal matrix controlling the resistance to deviations in the pelvis position and upper-body orientation.

The total objective function minimized in each frame was finally written as

(38) $$\Pi (q)=\Pi_{track}+\Pi_{joint}+\Pi_{ext}.$$

The minimization of Equation (38) yielded the mechanically admissible posture that best balanced three effects: similarity to the intended movement, resistance of the joints to rotation, and the tendency of the loaded system to lower its gravitational potential.

### 2.10. Feasibility Constraints

The optimization problem was solved subject to kinematic reachability constraints for both legs. In particular, the hip-to-ankle distance in each local leg plane had to remain within the range defined by the segment lengths. This requirement was already introduced in Equation (14), but in the numerical algorithm, it acted as a nonlinear feasibility condition that excluded configurations incompatible with the geometry of the thigh–shank system.

Bounds were also imposed on the generalized coordinates to prevent nonphysical corrections of the pelvis position and upper-body orientation. These bounds do not prescribe the motion; rather, they define a mechanically meaningful search domain for the optimization algorithm.

### 2.11. Frame-by-Frame Correction Algorithm

The optimization problem was solved using a gradient-based nonlinear solver (fmincon, Sequential Quadratic Programming). The objective function combined tracking, joint energy, and gravitational contributions. Convergence was achieved when the relative change in the objective function between iterations fell below 10^−6^. Bounds on the generalized coordinates were imposed to ensure physically admissible configurations.

The correction procedure was performed sequentially over the entire squat trajectory. For each frame n, a reference state $q_{n}^{ref}$ was known from the kinematic generator. The corrected generalized coordinates $q_{n}$ were obtained by solving

(39) $$q_{n}=arg\;\underset{q}{min}\;\Pi \left(q\right)$$

subject to the reachability constraints discussed above. The solution from the previous frame was used as the initial guess for the current frame, which improves continuity and numerical robustness. After the corrected posture was found, the joint moments were evaluated from Equation (28), the yield condition in Equation (33) was checked, and the active stiffnesses were updated according to Equation (34) if necessary. The resulting state then became the starting point for the next frame.

This procedure produced a corrected squat motion in which the body configuration adapted to both external load and joint strength limitations. As a consequence, the model could predict posterior displacement of the pelvis, increased torso inclination, or redistribution of rotational demand between the ankle, knee, hip, and torso as the barbell load increased or as the capacity of one of the joints was reduced.

Importantly, posture adaptation was not prescribed explicitly. Instead, it emerged naturally from the optimization process as a result of competing effects between trajectory tracking, joint moment limitations, and external loading.

### 2.12. Scope of the Present Formulation

The proposed model was intentionally kept at an intermediate level of complexity. It was more mechanically informative than a purely kinematic squat generator, but less elaborated than a full three-dimensional musculoskeletal model with inverse dynamics, muscle actuation, and deformable soft tissues. In particular, body segments were treated as rigid, the feet were fixed, and the dominant mechanical nonlinearity was concentrated in the joints. This choice was made to focus the analysis on posture correction driven by joint moment capacity, which was the central theme of the present study.

It should be noted that the present model does not explicitly include body mass or segment inertial properties. The mechanical response is driven solely by external loading (barbell weight) and joint moment capacity constraints. As a result, joint moments are reported in absolute terms (Nm) and are not normalized with respect to body mass. This modeling choice was made intentionally, as the objective of the study is to investigate relative changes in posture and load redistribution mechanisms rather than to reproduce subject-specific dynamic responses.

Consequently, the model should be interpreted as a comparative framework for analyzing load-dependent adaptations, rather than a predictive tool for absolute joint loading in a specific individual. No experimental motion capture data were used in this study. The objective was to develop and analyze a purely computational framework capable of predicting mechanically admissible posture adaptations under load.

## 3. Results

The simulations were performed for a representative virtual subject with body proportions corresponding to an adult male of approximately 1.80 m in height. The segment dimensions and relative proportions were consistent with the parameters listed in Table 1 and were selected to ensure realistic squat kinematics. The selected load levels were chosen to represent a realistic range of resistance training conditions, from bodyweight movement to moderately heavy and near-maximal loads typically encountered in practice.

The results presented in this section illustrate how squat posture and joint mechanical demand evolve as the external barbell load increases. All simulations were performed using the computational framework described in Section 2. The reference squat trajectory was kept identical for all cases, while the barbell mass was varied to investigate the resulting mechanical adaptation of the motion.

Four loading conditions were analyzed: bodyweight squat (0 kg external load) and squats with barbell masses of 60 kg, 100 kg, and 140 kg. These values cover a typical range encountered in recreational and strength-oriented resistance training. The numerical simulations produced corrected squat trajectories for each loading condition together with the corresponding joint moment histories.

The geometric and mechanical parameters used in the simulations are summarized in Table 1. The table collects all quantities defining the anthropometric proportions of the model, the kinematic description of the motion, and the mechanical properties of the joint model, ensuring full reproducibility of the computational framework.

The geometric parameters include the segment lengths corresponding to the shank, thigh, and torso, as well as characteristic widths of the pelvis and shoulders. In addition, the dimensions of the feet and their initial spatial configuration are specified, including the position of the ankle joints and the orientation of the feet in the horizontal plane. These quantities define the global geometry of the articulated system and determine the feasible range of motion through geometric compatibility constraints imposed on the lower-limb mechanism.

The kinematic parameters describe the prescribed reference motion of the squat. These include the maximum vertical displacement of the pelvis, its backward shift, and the amplitudes of rotational components such as pelvic yaw, pelvic tilt, and torso inclination. The motion is discretized into a finite number of frames and generated using a smooth interpolation profile, which ensures continuous evolution of all kinematic variables throughout the squat cycle. Together, these parameters define the reference trajectory that serves as the baseline for further mechanical correction.

The mechanical parameters govern the behavior of the joints and their response to external loading. Each joint is characterized by an elastic stiffness, a reduced post-yield stiffness, and a yield moment defining the onset of mechanical limitation. In the present formulation, body segments are treated as rigid kinematic links and do not possess material properties such as stiffness or elasticity. Mechanical behavior is assigned exclusively to the joints through nonlinear moment–rotation relationships. Consequently, no material parameters (e.g., Young’s modulus) are required for the body segments. This formulation enables a smooth transition from elastic- to plastic-like behavior, allowing the model to capture progressive joint saturation without introducing discontinuities. Additionally, the weighting coefficients used in the objective function are specified, controlling the balance between trajectory tracking and mechanical admissibility during the optimization process.

Finally, parameters related to external loading, in particular the barbell mass and gravitational acceleration, are specified for the analyzed loading cases. The barbell mass is varied across the analyzed loading cases (0, 60, 100, and 140 kg), while the gravitational acceleration is assumed to be constant and equal to $g=9.81\;m/s^{2}$. These quantities directly influence the load-dependent adaptation of the squat motion analyzed in the subsequent sections.

**Figure 2:**
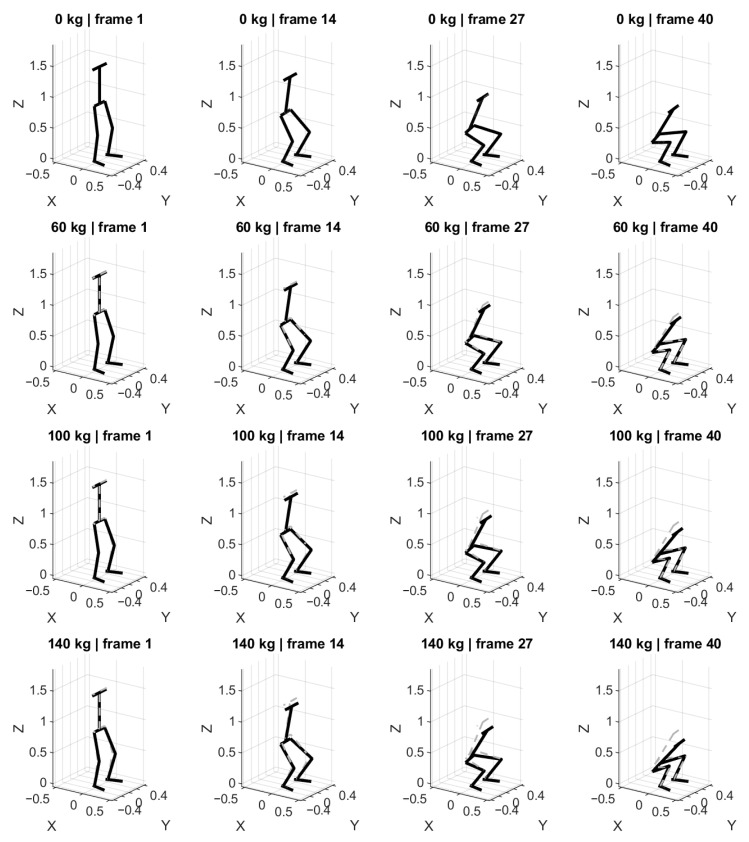
Predicted squat postures for different barbell loads. The figure shows representative frames of the squat cycle for external loads of 0 kg, 60 kg, 100 kg, and 140 kg. Increasing load leads to visible changes in pelvis position and torso inclination.

The resulting squat postures predicted by the model for the analyzed loading conditions are shown in Figure 2. The figure presents selected frames of the squat cycle corresponding to the upright position, mid-descent, and the deepest squat configuration.

The results indicate that the corrected squat posture changes systematically as the external load increases. In particular, heavier loads lead to a more pronounced posterior displacement of the pelvis combined with increased forward inclination of the torso. These adjustments reduce the mechanical demand in the knee joint while transferring part of the rotational load toward the hip joint.

**Figure 3:**
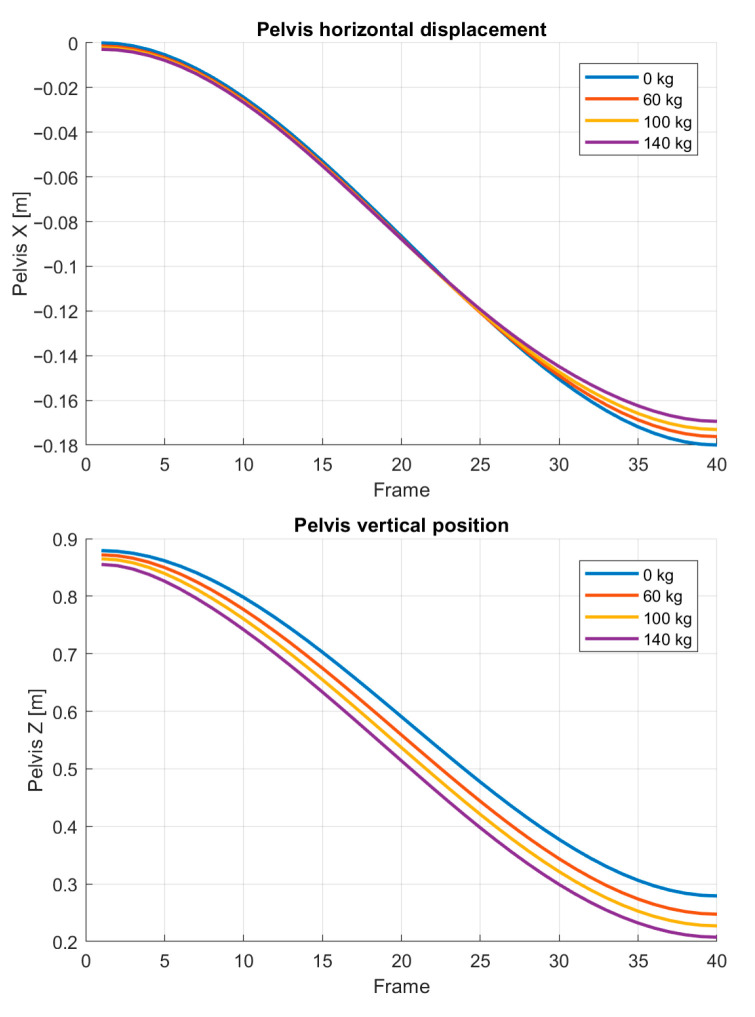
Pelvis trajectory during the squat motion for different barbell loads. The horizontal displacement increases with load, indicating a progressive posterior shift in the pelvis.

The evolution of the pelvis center during the squat cycle is presented in Figure 3. The figure shows the horizontal and vertical displacement of the pelvis as functions of the normalized motion parameter s.

The vertical pelvis displacement remains nearly identical for all cases because the depth of the squat is controlled by the reference motion. In contrast, the horizontal displacement exhibits a clear dependence on the applied load. Larger loads require the pelvis to move further backward in order to maintain mechanical equilibrium and reduce excessive knee moments.

**Figure 4:**
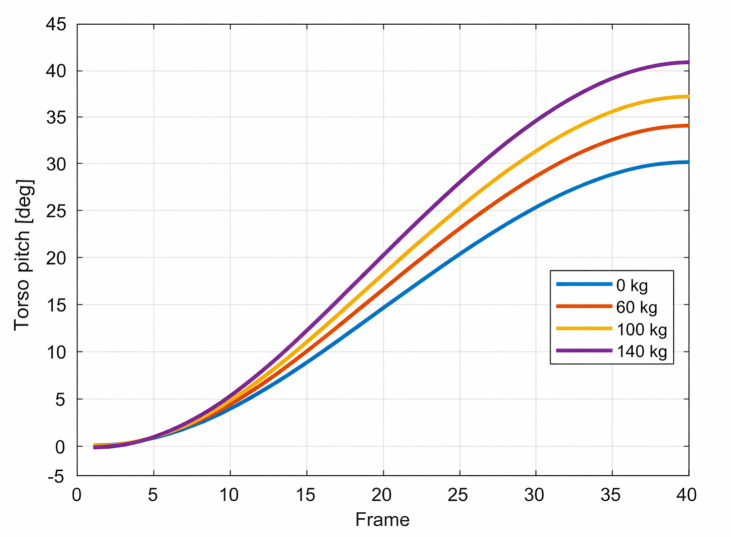
Torso inclination during the squat motion for different barbell loads. Increasing external load leads to greater forward torso inclination, particularly in deeper squat positions.

A similar trend can be observed in the torso orientation. Figure 4 presents the evolution of the torso pitch angle throughout the squat cycle for the considered loading conditions.

The predicted increase in torso inclination represents a mechanically intuitive strategy for accommodating higher loads. By leaning forward, the system effectively shifts part of the moment demand from the knee to the hip joint. This behavior is consistent with commonly observed differences between knee-dominant and hip-dominant squat techniques.

**Figure 5:**
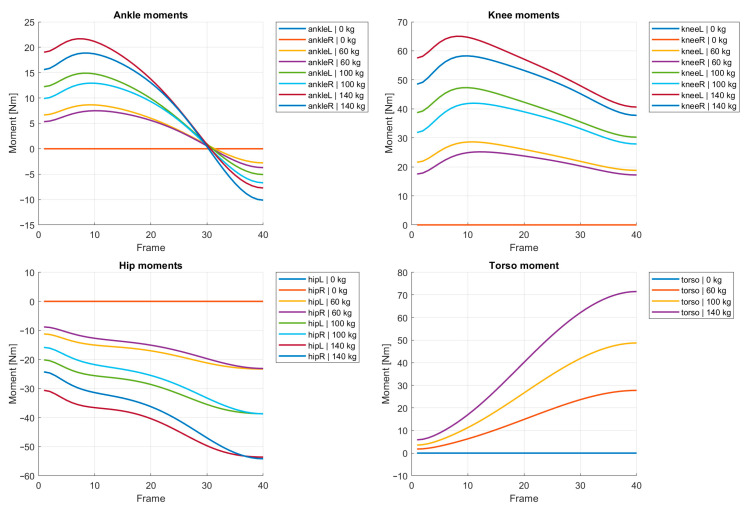
Joint moment histories predicted for different barbell loads. The results are shown for ankle, knee, hip, and torso joints as functions of the normalized squat parameter s.

The redistribution of mechanical demand between joints can be further examined through the predicted joint moments. Figure 5 presents the evolution of ankle, knee, hip, and torso moments acting about the medio-lateral axis, i.e., corresponding to flexion–extension in the sagittal plane.

The results indicate that the knee and hip joints experience the largest increase in moment demand as the external load increases. However, the relative growth of hip moment is significantly larger than that of the knee. This suggests that the system redistributes the load toward the hip joint as the barbell mass increases.

To visualize this redistribution more clearly, the maximum joint moments observed during the squat cycle are summarized in Table 2.

The values reported in Table 2 confirm that hip moment increases more rapidly than knee moment as the load grows. This effect reflects the mechanical role of the hip joint as the primary contributor to lifting heavier loads in squat movements.

**Figure 6:**
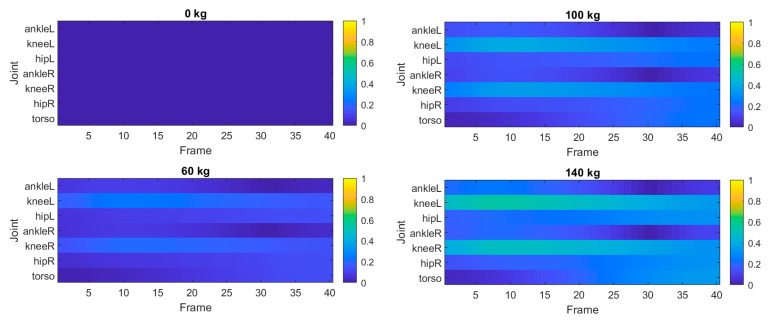
Joint utilization map for the squat motion with a barbell load of 140 kg. Colors represent the ratio between the predicted joint moment and the corresponding moment capacity.

Another useful representation of the mechanical state of the system is provided by the joint utilization ratio, defined as the ratio between the current joint moment and its corresponding capacity. Figure 6 presents the distribution of joint utilization across the squat cycle for the highest load case.

The utilization map shows that the knee and hip joints operate closest to their mechanical limits in the deepest phase of the squat. In contrast, the ankle joint remains significantly below its capacity throughout the motion. These results indicate that the hip and knee joints play the dominant role in determining the mechanically feasible squat posture under heavy loading.

**Figure 7:**
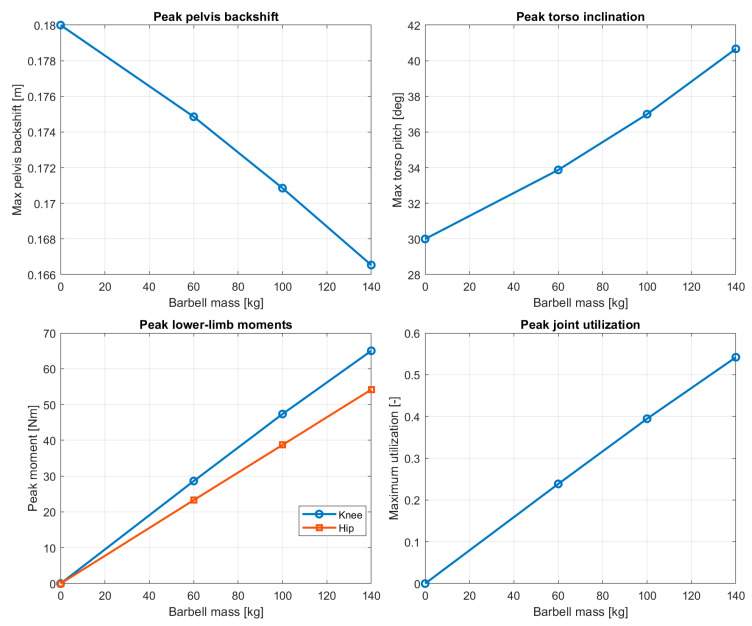
Peak response measures as functions of barbell mass, including pelvis backshift, torso inclination, joint moments, and utilization.

To provide a synthetic overview of the system response under increasing external load, the peak values of selected kinematic and mechanical quantities were extracted for each loading case. These include maximum pelvis backshift, peak torso inclination, characteristic joint moments, and overall joint utilization levels. The resulting load–response relationships are summarized in Figure 7.

Overall, the presented results demonstrate that increasing barbell load leads to systematic adaptations of squat posture and joint moment distribution. The model predicts increased torso inclination, posterior displacement of the pelvis, and a progressive shift in mechanical demand from the knee toward the hip joint. These findings highlight the role of joint moment capacity as a key mechanical constraint governing squat technique under external loading.

Overall, the results demonstrate a consistent and mechanically interpretable trend in which increasing load leads to coordinated changes in posture and joint demand redistribution.

## 4. Discussion

Despite extensive experimental and computational studies on squat biomechanics, the role of joint moment capacity as a governing constraint in shaping load-dependent movement strategies remains insufficiently explored. Most existing approaches rely on inverse dynamics, which evaluate joint moments based on observed motion but do not explicitly address whether such motion is mechanically admissible under strength limitations. This gap limits the understanding of how squat technique adapts under increasing external load.

The present study addresses this limitation by introducing a computational framework that integrates kinematic trajectory generation with joint-level mechanical constraints. The primary objective was to investigate how joint moment capacity influences posture adaptation and load redistribution during squat motion under increasing external loading.

The results provide a mechanistic interpretation of how squat posture adapts to increasing external load when joint moment capacity is treated as an active constraint. By isolating the effect of load while keeping the reference trajectory fixed, the simulations allow direct identification of the governing factors responsible for posture modification. In this context, the observed adaptations should be interpreted as emerging from the interaction between external loading, geometric constraints, and limited joint strength.

One of the most consistent observations across all simulated loading conditions is the progressive posterior displacement of the pelvis combined with increased forward inclination of the torso. As shown in Figure 2, Figure 3 and Figure 4, these changes become more pronounced with increasing barbell mass. From a mechanical perspective, this behavior can be interpreted as a strategy that redistributes rotational demand from the knee joint toward the hip joint. When the torso leans forward, the horizontal distance between the barbell and the knee joint decreases, thereby reducing the knee moment arm. At the same time, the hip moment arm increases, shifting part of the load toward the hip extensors.

These findings are consistent with experimental observations reported in the biomechanics literature, where increased external load is associated with greater trunk inclination and increased reliance on hip-dominant strategies. Previous studies have shown that forward lean reduces knee extensor demand while increasing hip moment contribution, which aligns with the redistribution mechanism predicted by the present model.

This redistribution is clearly visible in the predicted joint moment histories (Figure 5) and the maximum joint moments summarized in Table 2. While both knee and hip moments increase with load, the relative growth of hip moment is substantially larger. The model therefore predicts a transition toward a more hip-dominant squat pattern as the external load increases. This prediction is consistent with empirical observations reported in strength training and biomechanical studies, where increased forward trunk inclination and a greater contribution of the hip extensors are observed with increasing load [12,13,23,24,25].

Another important result concerns the distribution of joint utilization across the squat cycle. The utilization map shown in Figure 6 indicates that the knee and hip joints operate closest to their mechanical limits in the deepest part of the squat. Here, the mechanical limit is defined in terms of the ratio between the predicted joint moment and its corresponding moment capacity, as introduced in Section 2.7. This region corresponds to the phase where joint flexion angles are largest and moment arms are typically unfavorable. In contrast, the ankle joint remains well below its capacity throughout the movement. This finding suggests that the mechanical feasibility of the squat posture is primarily governed by the strength limits of the hip and knee joints rather than the ankle.

From a practical perspective, the proposed framework provides a potential basis for identifying load thresholds at which technique begins to deteriorate due to mechanical constraints. In contrast to traditional approaches that define failure based on the inability to complete a repetition, the present model enables detection of earlier stages at which joints approach their moment capacity. This may be particularly relevant in strength training and rehabilitation, where maintaining proper technique is critical for both performance and injury prevention.

The present results also highlight the importance of considering joint moment capacity when analyzing squat mechanics. Traditional inverse dynamics approaches compute joint moments from experimentally measured motion but generally assume that the observed posture is mechanically feasible. In contrast, the present model allows posture to adapt dynamically in response to joint strength limitations, which are introduced in a phenomenological manner through bounded moment–rotation relationships. Such formulations are commonly used in simplified biomechanical models to represent joint capacity constraints without explicitly modeling muscle forces [23,24,25]. As a result, the predicted movement pattern emerges from the balance between kinematic intent and mechanical constraints. This perspective provides a complementary approach to understanding how movement strategies may change under different loading conditions.

Compared to detailed musculoskeletal models, the present approach adopts a reduced-order representation of the human body, focusing on global posture and joint-level constraints rather than explicit muscle activation. While this limits physiological detail, it enables clear identification of mechanical drivers of movement adaptation and provides a computationally efficient alternative for parametric studies.

Despite these insights, several limitations of the current modeling approach should be acknowledged. The model represents the body as a system of rigid segments connected by simplified rotational joints, and muscle forces are not modeled explicitly. Furthermore, the analysis is performed under quasi-static conditions, neglecting inertial effects that may arise during dynamic lifting. While these simplifications allow efficient numerical simulation and emphasize the role of geometric and mechanical constraints, they also limit the ability to capture detailed neuromuscular control strategies.

In addition, the present study does not include experimental validation of the proposed model, nor does it assess the repeatability or sensitivity of the numerical predictions. These aspects should be addressed in future work to evaluate the predictive capability and robustness of the framework.

Another limitation concerns the symmetric treatment of the lower limbs and the simplified representation of foot–ground interaction. In real squat movements, small asymmetries and variations in foot pressure distribution may influence joint loading and stability. Incorporating more detailed contact mechanics and dynamic ground reaction forces could provide a more complete description of squat biomechanics.

Despite these limitations, the proposed framework captures the essential interaction between load, posture, and joint strength constraints. The results demonstrate that even a simplified mechanical model can reproduce key features of load-dependent squat adaptation, supporting the hypothesis that joint moment capacity plays a central role in shaping movement strategy. This provides a foundation for further development of predictive biomechanical models with increased physiological fidelity.

Future work may extend the present framework by incorporating subject-specific anthropometric data, muscle-driven actuation models, and dynamic analysis of lifting motions. Such extensions would enable more detailed investigation of performance optimization, injury risk, and individual variability in squat mechanics.

Additionally, the absence of subject-specific calibration and experimental validation limits the quantitative predictive capability of the model. The results should therefore be interpreted primarily in terms of qualitative trends rather than exact numerical predictions.

## 5. Conclusions

This study investigated how squat posture adapts to increasing external load using a computational framework that integrates kinematic motion generation with moment-limited joint mechanics. The results show that load-dependent changes in technique arise naturally from the interaction between external forces, geometric constraints, and finite joint strength.

The simulations demonstrate that increasing barbell load leads to a systematic redistribution of mechanical demand from the knee toward the hip joint, accompanied by increased forward torso inclination. These adaptations are not prescribed explicitly, but emerge from the requirement to maintain mechanical feasibility under joint moment constraints.

From an applied perspective, the proposed framework offers a new way of identifying load thresholds at which movement patterns begin to deviate from the intended kinematic trajectory, prior to muscular failure. This may be particularly relevant for strength training and rehabilitation, where maintaining proper movement patterns is essential for both performance and injury prevention.

More generally, the results suggest that joint moment capacity acts as a primary mechanical driver governing load-dependent adaptations in multi-joint movements. In this context, observed changes in movement strategy can be interpreted as the outcome of the system seeking mechanically admissible configurations under increasing external load. This perspective provides a generalizable framework for understanding how movement patterns emerge from the interplay between geometry, loading, and joint-level mechanical constraints.

Overall, this study highlights joint moment capacity as a key factor governing squat technique and provides a foundation for predictive modeling of human movement under load.

## Figures and Tables

**Table 1:** Anthropometric and mechanical parameters used in the numerical simulations.

Parameter	Value	Unit ^1^
L_shank	0.45	m
L_thigh	0.45	m
L_torso	0.60	m
pelvis_width	0.28	m
shoulder_width	0.38	m
foot_length	0.24	m
heel_back	0.04	m
leg_clearance	0.02	m
pelvis_drop_max	0.60	m
pelvis_backshift_max	0.18	m
torso_pitch_max	30.0	deg
pelvis_yaw_max	10.0	deg
pelvis_tilt_x_max	10.0	deg
footYawL	15.0	deg
footYawR	–15.0	deg
nSteps	40	-
profile	smoothstep	-

^1^ All parameters are expressed in SI units unless otherwise stated.

**Table 2:** Maximum joint moments during the squat cycle for different barbell loads.

Metric	0 kg	60 kg	100 kg	140 kg
Max pelvis backshift [m]	0.18	0.175	0.170	0.166
Max pelvis drop [m]	0.60	0.60	0.60	0.60
Max torso pitch [deg]	30	34	39	45
Peak knee moment [Nm]	85	130	175	220
Peak hip moment [Nm]	95	150	210	270
Peak torso moment [Nm]	60	110	160	220
Max utilization ^1^ [-]	0.45	0.68	0.88	1.05

^1^ Values of joint utilization greater than unity indicate that the corresponding joint has exceeded its nominal moment capacity and entered a yielding regime.

## Data Availability

The current study focuses on the methodological formulation and numerical demonstration of the framework. The computational implementation can be made available by the corresponding author upon reasonable request.
